# The ReThink study: a 3-arm parallel randomized trial of cognitive bias modification, with and without adherence promotion, for adolescent anxiety disorder: trial design and protocol

**DOI:** 10.1186/s12888-019-2296-z

**Published:** 2019-10-22

**Authors:** Shelley Reetz, Gregory Clarke, Robin Weersing, Nader Amir, John Dickerson, Frances L. Lynch, Michael C. Leo, Andreea M. Rawlings, Mi H. Lee, Sara Gille

**Affiliations:** 1 Kaiser Permanente Center for Health Research, 3800 North Interstate Avenue, Portland, OR 97227 USA; 2 San Diego State University (SDSU), 5500 Campanile Drive, San Diego, CA 92182 USA; 3 Center for Understanding and Treating Anxiety, San Diego State University (SDSU), 6386 Alvarado Ct., Suite 301, San Diego, Ca 92120 USA

**Keywords:** Anxiety disorder, Adolescent, Cognitive bias modification, Randomized controlled trial

## Abstract

**Background:**

Anxiety disorders are the most common mental health problem among youth, contribute to reduced quality of daily life, and are associated with high rates of comorbidity. However, treatment rates for anxiety are very low, causing a sizeable treatment gap. There is an immediate need to identify treatment interventions that are effective, affordable, and can be delivered easily to the youth population. Cognitive Bias Modification (CBM) is one potentially effective intervention that could reach youth on a large scale, especially when self-administered at home. Thus, we aim to assess the benefit of CBM to treat youth anxiety. Further, we aim to test whether adding an adherence promotion (AP) component to the CBM intervention can improve outcomes, and whether CBM delivered both with and without the AP component is cost effective.

**Methods:**

This is a 12-month randomized controlled trial (RCT) conducted within an existing healthcare system. Potentially eligible youth (ages 12 to 17) will be identified by reviewing the electronic health record (EHR) for clinical anxiety diagnoses, which are then confirmed via research interview. We aim to enroll 498 participants and randomize them 1:1:1 to one of three arms: Arm 1 is a Low-Ratio version of the CBM program (nearly identical to the other CBM versions, but minimally effective); Arm 2 is a High-Ratio “active” CBM program; and Arm 3 is the High-Ratio CBM program with an added AP component. Participants will complete assessments at baseline, 1-, 3-, 6- and 12-months post-baseline. Youth in all three arms will self-administer the CBM program at home and will be asked to complete twelve intervention sessions over a four-week period. Arm 3 participants (High-Ratio CBM + AP) will also receive up to four telephone calls from phone coaches during the intervention period to provide technical assistance, encouragement, and motivational enhancement to increase adherence. The primary clinical outcome will be anxiety remission at 6-month follow-up.

**Discussion:**

This study protocol describes the method and design for an RCT to test whether self-administered CBM both with and without adherence promotion can be an effective at-home treatment for anxious youth.

**Trial registration:**

ClinicalTrials.gov: NCT02156531, First Posted June 5, 2014.

## Background

Approximately 1 in 3 adolescents meet criteria for an anxiety disorder by age 18, making anxiety disorders the most common mental health problem among youth [1]. These disorders interfere with daily life, have high rates of medical and psychiatric comorbidity [1, 2], are associated with high health care costs [3, 4], and are comorbid with substance abuse, depression and functional impairment in adulthood [5]. Additionally, anxiety has the lowest treatment rate of any mental disorder in childhood and adolescence [6, 7]; less than one-third of youth identified with anxiety received any intervention [8]. This disparity between high prevalence and low treatment makes addressing this “care gap” a significant public health priority.

What treatments are known to be effective for youth anxiety disorder? While there is a large body of evidence supporting the efficacy of both cognitive behavioral therapy (CBT) and medications such as selective serotonin reuptake inhibitors (SSRIs) for anxiety disorders, there are significant patient, intervention, and organizational barriers to youth receiving these evidence-based treatments (EBTs). Youth experiencing anxiety disorders typically have multiple psychosocial stressors and comorbidities [9] that inhibit active engagement in CBT. CBT demands a substantial time commitment on the part of both patient and therapist, is expensive to deliver, and is not a widely available treatment option due to the lack of qualified therapists available (particularly in rural areas). Although antidepressant (AD) medications are accessible and easier to administer, significant barriers leading to treatment nonadherence have been well documented, such as concerns about adverse effects, fears of dependence, and the often weeks- or months-long delay before therapeutic response [10]. As a result, there is an immediate, unmet need to identify and disseminate effective treatment interventions that are accessible, affordable, have few or no negative side effects, and can be delivered easily to the youth population.

One potentially effective anxiety intervention that could reach affected youth on a large scale is computerized Cognitive Bias Modification (CBM). The role of cognitive bias in the development and maintenance of anxiety has been well established from basic cognitive research [11, 12]. Biased attention towards real or perceived threat is associated with anxiety due to hypervigilance, rumination, and inaccurate appraisal of risks. Past research has shown that this biased attention towards negative rather than positive or neutral stimuli increases anxiety, and is related to generalized anxiety, social phobia, and trait and state anxiety [13, 14]. CBM is one of several related treatments that aim to retrain individuals to focus attention on positive or neutral stimuli, rather than negative or threatening stimuli. CBM is designed to adjust attentional bias at this early stage of processing, when patients are engaged in hypervigilance and over-attention to threatening cues. At this early information processing stage, training to focus attention away from threat has been shown in early studies to reduce anxiety symptoms [15–18]. The effects of CBM delivered under optimal conditions have been shown to be similar in magnitude to CBT and SSRI treatment for anxiety [15], and shows promising results in reducing anxiety in adult populations [18–22].

In youth populations, prior studies of CBM have shown significant symptom improvement in both anxiety and depression when delivered in a lab-based or residential treatment facility setting [15, 23–25]. However, because CBM is a self-administered computerized program, one advantage is the ability to disseminate it to patients at home, as it has a lower cost and time burden to implement than in-person administration. However, few studies have examined the effect of CBM in a real-world setting. Therefore, it is critical to test this promising treatment in conditions similar to those in which it could be eventually disseminated, as it is sometime the case that intervention effects observed in highly controlled trials diminish when evaluated in real world circumstances [26].

At least one study examined the efficacy of CBM delivered at home [22]. In that study, Carlbring and colleagues examined self-administered internet-based CBM in a home setting in 79 adults with social anxiety. These researchers did not find a significant difference between groups at post-treatment and 4-month follow-up. Neubauer and colleagues [27] also failed to find a benefit of Internet, home-delivered CBM versus the control condition at 4-month follow-up among their study of individuals with social phobia (*n* = 56).

One explanation for the weaker effects in self-administered CBM may be poor patient adherence when the intervention is delivered at home when compared to staff-facilitated lab settings. As a higher number of completed training sessions is associated with larger effect sizes [28], and in some studies, explicit staff-administered instructions for completing the CBM sessions may improve efficacy [29], another possible explanation for the lower efficacy of self-administered CBM may be reduced dose.

While most previous research of self-administered CBM has been conducted with adults, a number of studies have examined self-administered CBM among anxious youth [23, 24]. However, the effectiveness of self-administered CBM among youth remains to be tested on a large-scale basis. Therefore, to address these gaps in the literature, we aim to conduct a large efficacy-effectiveness trial within an existing health-care system to assess the benefit of computerized CBM to treat youth (ages 12–17) with one or more clinical and research-confirmed anxiety diagnosis. We identify this trial as having an efficacy-effectiveness design because we implement rigorous research inclusion criteria and outcome assessment, and will examine CBM mediational mechanisms (efficacy features), but at the same time we enroll participants who are seeking routine clinical care for anxiety in a large active healthcare system, have clinical anxiety diagnoses, and the intervention is self-administered (effectiveness features). We aim to test whether a telephone-based adherence promotion (AP) program can improve adherence and CBM dose to the levels obtained when CBM is delivered in laboratories. To our knowledge, this study will be the first to test this added component. The AP protocol consists of 1 to 4 brief telephone calls to provide explicit instructions to complete the sessions, offering additional support and motivational enhancement techniques [30–32] when warranted, and problem-solving barriers to completing the sessions.

We will conduct a randomized controlled trial of three arms, each corresponding to different versions of the CBM program: Low-Ratio CBM (Arm 1; a control/low dose version of CBM), High-Ratio “conventional” CBM (Arm 2), and High-Ratio CBM with AP (Arm 3). Ratio refers to the proportion of CBM trials in each session that are intended to retrain negative bias (details below). All versions of the intervention will be self-administered on participants’ home computers, as this would be the setting of eventual dissemination of this intervention. We hypothesize that the two High-Ratio CBM arms (Arms 2 and 3) will lead to greater rates of remission for anxiety diagnoses and greater improvement of symptoms and functioning, compared to the Low-Ratio CBM condition (Arm 1). Additionally, we hypothesize that the High-Ratio CBM with AP arm (Arm 3) will result in greater rates of anxiety remission and symptom improvement, compared to the High-Ratio CBM-only arm (Arm 2).

If we find that self-administered computerized CBM is an effective treatment option in real world settings, it will be important to consider whether broad dissemination and implementation is feasible. To evaluate this, we will compare the costs and outcomes of delivering High-Ratio CBM both with and without the adherence promotion component in an incremental cost effectiveness analysis. Specifically, we will examine whether High-Ratio CBM with AP will have benefits that warrant the additional cost of implementation (i.e., the additional labor costs associated with the AP portion of the intervention).

## Methods

### Design

This study is approved by the Kaiser Permanente Northwest (KPNW) Internal Review Board (IRB) in Portland, Oregon. This study is a randomized controlled trial (RCT), with participants blinded to the type of CBM they receive (low vs. high ratio) but not blinded regarding receipt of AP contacts. All evaluators are blinded to participant study condition.

Participants will be randomized via computer-programming to one of three arms in a 1:1:1 ratio, stratified by age at recruitment (< age 15 vs. > = age 15) and minority status (white non-Hispanic vs. all others). Assignment will be conducted with computer-generated random block sizes of 6 or 9, masked from study staff.

Arm 1 is a Low-Ratio version of the CBM intervention, nearly indistinguishable from the other CBM version but with a lower proportion of trials that shift attention to the neutral stimuli (details below). Arm 2 is a High-Ratio CBM program, with a higher proportion of trials shifting attention to the neutral stimuli. Arm 3 is the same High-Ratio CBM program but with a component consisting of adherence promoting (AP) telephone calls from a study phone coach, from 1 to 4 total as needed. All CBM programs are self-administered on participants’ home computers.

The primary clinical outcome will be diagnostic remission at 6-month follow-up from all anxiety diagnoses present at baseline, as confirmed by the Anxiety Disorders Interview Schedule (ADIS) (see below). The secondary clinical outcomes will be the PARS, SCARED, attention bias, depression (PHQ-9), psychopathology severity (CGI-S), and psychosocial functioning (CGAS). We will also determine whether there are differences in treatment acceptability (PAQ) and satisfaction (CSQ-8). Another secondary outcome will be cost-effectiveness of the CBM intervention, as measured in incremental cost per anxiety-free-day (AFD) and cost per quality adjusted life year (QALY). Additional analyses will test whether baseline CGI-S, PHQ-9 (depression), severity of cognitive bias (ADP), gender, socio-economic status, and race/ethnicity moderate the effect of the intervention on the primary outcome. Additionally, we will test whether attention bias mediates the effect of the intervention on the 6-month remission rate and anxiety outcomes (PARS and SCARED). Lastly, we will examine whether CBM process variables (i.e., number of trials [dose], acceptable error rates, reaction time) is associated with CBM outcomes.

### Population and recruitment

Youth between the ages 12 to 17 years old will be enrolled from the KPNW healthcare organization, a large, non-profit health maintenance organization in the Pacific Northwest region of the USA. We aim to enroll a total of 498 participants in the study.

Potentially eligible youth will be identified by reviewing the healthcare organization electronic health record (EHR) for anxiety diagnoses (general anxiety disorder [GAD], anxiety disorder unspecified, or social phobia [SP], and/or separation anxiety). These are not qualifying criteria themselves, but a preliminary case-finding stage that identifies youth who are likely study eligible whom we will approach for recruitment. This initial EHR stage is meant to improve case-finding efficiency. Teen and parent participants will be recruited by mail, with a follow-up telephone call. Interested families will complete a brief, initial phone screening interview to determine if they meet preliminary inclusion and exclusion criteria. If preliminary eligibility criteria are met, parents and youth will give consent to participate in the full baseline assessment.

Inclusion criteria for this study include: age between 12 and 17 years old at enrollment; a research anxiety diagnosis confirmed by the ADIS at the baseline interview (see below); youth and parent able to complete assessments in English; youth access to a Windows home computer with Internet access. Exclusion criteria include: youth diagnosis of psychotic disorder; significantly impairing learning disorder or processing problem, such that completion of study procedures would be difficult; ADHD (except if symptoms are stable and controlled by medication for at least one month prior to enrollment); or youth primary complaint of condition other than anxiety. Comorbid diagnoses leading to study exclusion will be confirmed by reviewing the EHR.

We considered whether to require a minimum level of cognitive bias at baseline as an inclusion criterion. Based on the literature at the time of our proposal, higher pre-treatment levels of negative bias were associated with improved outcomes [33], although this question has been little tested in anxious youth. Ultimately, we elected to not require a minimum bias level, for several reasons. First, the literature on this issue is suggestive but not definitive, especially for youth. Second, the effectiveness nature of this trial argues for the broadest inclusion possible with the most representative sample. Third, including all youth regardless of baseline bias provided an opportunity to test this relationship in a very large sample.

### Assessments

After the preliminary screening call, interested and potentially eligible participants will complete a baseline interview where final eligibility is determined. All measures at baseline will be administered to parent and youth participants over the phone, prior to randomization. See Table 1 for the assessment schedule.

**Table 1:** Assessment Schedule

Measures	Administration time (mins)	Respondent	Youth or Parent Sx	Timepoints*					
				S	T0	T1	T2	T3	T4
Phone eligibility screen	15	Y P	Y	x					
Youth Anxiety measures									
Anxiety Dx Interview Schedule-Child (ADIS-C)**	50	Y P	Y		x			x	x
Pediatric Anxiety Rating Scale (PARS)	15	Y P	Y		x	x	x	x	x
Screen Child Anxiety Relate Emotional Dx (SCARED)	10	Y P	Y		x	x	x	x	x
Child Avoidance Measure–Self Report (CAMS)	5	Y	Y		x	x	x	x	x
Child Avoidance Measure–Parent Report (CAMP)	5	P	P		x	x	x	x	x
Depression measures									
Patient Health Questionnaire (PHQ-9)	10	P	Y		x	x	x	x	x
Patient Health Questionnaire (PHQ-9) (Parent self-report)	5	P	P		x	x	x	x	x
Other psychopathology, improvement measures									
Clinical Global Improvement –Severity (CGI-S)	5	Y	Y		x				
Clinical Global Improvement –Improvement (CGI-I)	–	Y	Y			x	x	x	x
Psychosocial functioning, quality of life measures									
Children’s Global Adjustment Scale (CGAS)	–	Y	Y		x	x	x	x	x
Healthcare services, economic measures									
Child & Adolescent Services Assessment (CASA)	5–10	Y P	Y			x	x	x	x
EQ5D	5	Y P	Y		x	x	x	x	x
Other measures									
Demographics	5	P	–		x				
State Trait Anxiety Inventory (STAI) – State	5	P	P		x	x	x	x	x
Peterson Pubertal Development Scale (PDS)	5	Y	Y		x				
Insomnia Severity Index (ISI)	5	Y	Y		x	x	x	x	x
Participant Acceptability Questionnaire (PAQ)	5	Y P	–			x			
Client Satisfaction Questionnaire (CSQ-8)	5	Y P	Y			x			
U Rhode Island Change Assessment (URICA-S)	5	Y	Y		x	x			
Instrumental Variables (IV)	5	Y	Y		x				
CBM session review	5	Y	Y			x			
Treatment adherence and process data	–	–			x	x	x	x	x

*Timepoints: S = Screening; T0 = Baseline; T1 = 1-month follow-up; T2 = 3-month follow-up; T3 = 6-month follow-up; T4 = 12-month follow-up

** Primary outcome, ADIS anxiety and depression modules only

**health care utilization data obtained from the HMO administrative data system

Dx = diagnoses/disorders, Sx = symptoms, Y=Youth, P=Parent, I=Interviewer

**Fig. 1:**
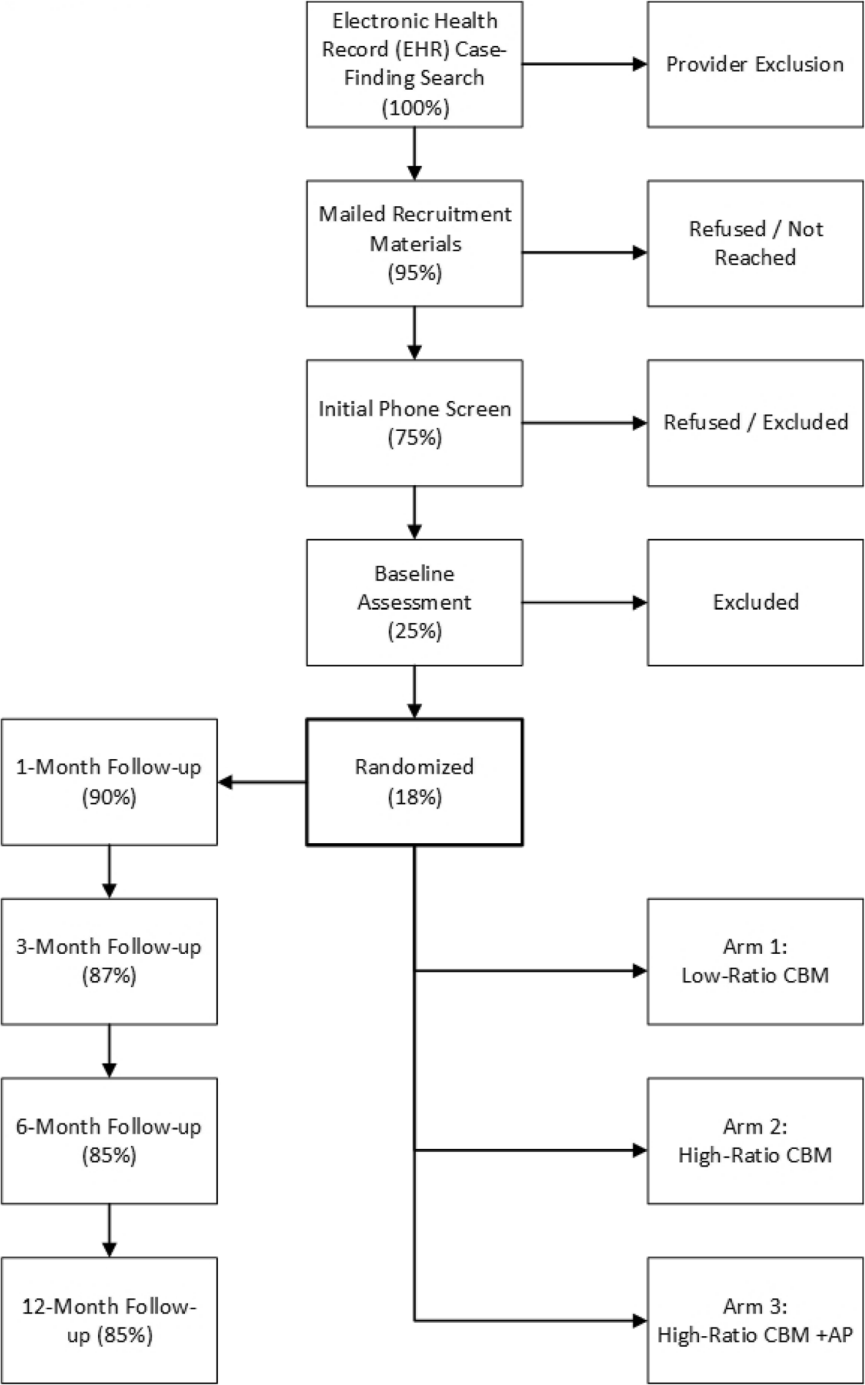
Participant Flow Diagram

Each assessment wave will take between 1 and 2 h each for youth and parents to complete. Youth and parent participants will complete four follow-up assessments: “post-treatment” at the end of the active intervention period one month after enrollment; and 3-month follow-up, 6-month follow-up, and 12-month follow-up. Youth will be asked to complete a single CBM session on their home computer at the time of each follow-up assessment, to measure negative bias rather than to reactivate the intervention. Details on the study design and participant flow are displayed in Fig. 1. Some measures (ADIS, PARS, CSSRS, CGAS, CGI-S/CGI-I, and demographics) will be administered by research evaluation staff over the phone, while the remaining self-report measures will be completed online by the parent and/or youth through a study web portal. In some cases when participants are unable or unwilling to complete the phone interview or online assessment, they may be mailed a survey packet to complete at home instead.

To promote adherence and follow-up completion participants receive Amazon gift certificates for completing baseline and each planned follow-up. Assessment staff made multiple calls to ppts to schedule or follow-up on any missed assessments.

Research evaluation staff will be blinded to CBM allocation and whether they receive AP calls. We have procedures to reduce the chance that research evaluation staff are made aware of subjects’ status (i.e., data sent from the participant’s performance on the CBM program will be sent directly to the programmers’ server and kept separate from the rest of the study data).

### Measures

#### Youth anxiety

The **Anxiety Disorders Interview Schedule for Children (ADIS-C)** [34, 35] is a semi-structured interview developed to identify the presence, severity, and level of interference of anxiety disorders in children aged 6–18 years old. It will be administered to youth and parents to yield Diagnostic and Statistical Manual of Mental Disorders (DSM) diagnoses of generalized anxiety disorder (GAD), social phobia (SP), and/or separation anxiety diagnoses needed to qualify for participation in the study. The trial primary outcome was also derived from this ADIS assessment: anxiety diagnosis remission at the 6-month follow-up. The ADIS will only be administered at the baseline, 6-month, and 12-month interviews. Before conducting ADIS interviews, staff must reach a criterion of 100% diagnostic correspondence with a senior staff on two consecutive interviews. All interviews will be digitally recorded; a random 10% of baseline assessments completed by each evaluation staff will be re-rated by a senior staff member.

The **Pediatric Anxiety Rating Scale (PARS)** [36] is a secondary outcome measure. This 50-item measure integrates youth and parent report of the severity of anxiety symptoms, as well as related functional impairment. Several versions of PARS scoring are available and used in the literature. To allow for comparison with other similar studies, we will calculate a total PARS score as the sum of items 2–7, with a total score ranging from 0 to 30 [37]. The PARS has high inter-rater reliability, fair internal consistency and test-retest reliability, convergent and divergent validity, and sensitivity to treatment effects [36, 38].

The **Screen for Child Anxiety Related Emotional Disorders (SCARED)** [39] is a 41-item measure of anxiety symptoms with youth- and parent-reported versions, with scores of > 25 indicating significant anxiety (total range 0 to 82). The SCARED has been validated in diverse clinical and community samples, with good internal consistency, and has detected clinical improvement in randomized clinical trials of treatment for youth anxiety [38]. We will examine the SCARED as a secondary outcome in this trial.

The **child avoidance measure – self (CAMS) and parent (CAMP)** [40] is an 8-item measure of a child’s tendency to avoid behavior that may cause fear, worry, or anxiety. Children are prompted with “When I feel scared or worried about something …” (or parents “When my child is faced with something that makes him/her feel scared or worried …” ) and asked to indicate their responses on each item using a 4-point Likert-type scale (0 = almost never; 1 = sometimes; 2 = often; 3 = almost always). Items are intended to cover passive avoidance, delay, and active refusal.

#### Depression

Youth and parents will both report on youth depression symptomatology on the **Patient Health Questionnaire (PHQ-9)** [41, 42] which yields depression symptom totals and a provisional DSM diagnosis. Parents will also complete the PHQ-9 to report on their own symptoms of depression. The PHQ-9 will be investigated as a secondary outcome and as a potential moderator of CBM effects.

#### Other psychopathology, improvement

We will assess overall clinical status with the 7-point **Clinical Global Impression (CGI)** Severity and CGI-Improvement scales [42]. The CGI provides a brief assessment of the patient’s overall psychiatric illness before and after initiating a medication or treatment. The CGI-S measures the severity of illness and will be assessed at baseline. At follow-up, the CGI-I will be used as a complementary measure to assess improvement in psychiatric illness after initiating treatment. A CGI-I score of < 2 (very much or much improved) is considered a response to treatment.

#### Psychosocial functioning, quality of life

The **Children’s Global Adjustment Scale (CGAS)** [43] is an adaptation of the Global Assessment Scale (GAS) and will be used to assess psychosocial functioning**.** Research evaluation staff will rate the 100-point CGAS where scores above 70 indicate normal functioning, and scores less than 60 typically indicate the need for treatment.

#### Healthcare services, economic measures

Health Maintenance Organization (HMO) TAU healthcare services, including medications, will be obtained from the HMO **Electronic Health Record (EHR)** system through 12-month follow-up.

In order to capture any services youth may receive outside the HMO, we will administer the **Child and Adolescent Services Assessment (CASA)** [44]. The CASA is a youth- and parent-report instrument designed to assess the use of services by children ages 8 years to 18 years. The CASA includes 31 settings covering inpatient/outpatient visits, medications, and informal services provided by a variety of child-serving providers and sectors, and will be administered to collect data regarding non-HMO services. This instrument collects information on whether a service was ever used and more detailed information (length of stay/number of visits, focus of treatment) on services used in the recent past.

The **EQ-5D** [45, 46] is a 5-item measure that provides a single index value for health-related quality of life. Respondents rate their health state in five domains: mobility, self-care, usual activities, pain/discomfort, and anxiety/depression. It has good performance properties with anxiety disorders [47–49] and with children with a variety of health conditions [50]. The EQ-5D will be administered to parents and youth, to report on youth symptoms.

#### Other measures

**Demographic information** will be obtained at baseline: youth and parent age, birthdate, sex, race, ethnicity, religion, school status, health status, parent marital status, family composition (i.e., parent’s marital status), and socio-economic status (i.e., family income).

The **State Trait Anxiety Inventory (STAI)** [51] is a 40-item measure that assesses current and general feelings of anxiety, completed by parents about their own anxiety. There are two subscales, the State Anxiety Scale (STAI-S), and the Trait Anxiety Scale (STAI-T). The STAI-S will be administered to parents and asks how they feel “right now” or “at this moment.” The 20 items include measures of apprehension, tension, worry, and nervousness, as well as measures of calmness, security, and confidence. Each item is scored on a 1–4 scale and all items are scored such that higher values indicate greater anxiety. A total score ranging from 20 to 80 is created by summing all 20 items.

The **Peterson Pubertal Development Scale (PDS)** [52–54] is a self-administered, validated scale for pubertal development, including growth, changes in body hair, skin, and voice (for boys) or breast development (for girls). We will administer the abbreviated 5-item version. Each item is scored from 1 (developmental characteristic/sign not yet begun) to 4 (characteristic seems complete).

The **Insomnia Severity Index (ISI)** [55, 56] is a brief 7-item instrument that measures insomnia perception over the last month. It will be administered to the youth participants. The items comprise severity (difficulty falling asleep, staying asleep, and waking), satisfaction with current sleep pattern, interference of daily function, noticeability to others, and worry/distress caused by the sleep problems. Each item is scored on a 5-point Likert scale (range 0 to 4), with total score ranges from 0 to 28.

The **Participant Acceptability Questionnaire (PAQ)** is a youth- and parent-reported measure of the intervention acceptability and credibility, adapted from similar scales used by the Research Units on Pediatric Psychopharmacology. The PAQ asks about burden (e.g., boredom), beliefs and expectations about the intervention, and task comprehension.

The **Client Satisfaction Questionnaire (CSQ-8)** [57] is a measure of healthcare satisfaction with services received. Each item is scored from 1 to 4, for a total score ranging from 8 to 32, with higher scores indicating greater satisfaction with services. The CSQ will be given to youth and parents and will be a secondary outcome.

The **University of Rhode Island Change Assessment (URICA)** [58] is a 32-item readiness for change questionnaire. For this study, we used a shorter 12-item version, with statements describing how a person might feel when starting therapy or approaching problems in their lives. Youth participants indicate the extent to which they tend to agree or disagree with each statement. The URICA will be administered to youth participants.

**Instrumental Variables (IVs)** are measures that may permit an estimate of dose effect that is less biased than raw dose-outcome analyses [59, 60]. Candidate IVs need to be highly predictive of CBM dose but unrelated to unobservable characteristics that would confound estimates of the effect of CBM on outcomes. IVs will be collected at baseline and will include the characteristics of environment (e.g. privacy, location) where the youth plans to use the computer for the intervention, and the current use of electronic devices (e.g. hours of video games played per day).

### Interventions

Youth in all three arms will download one of two versions of the CBM program, depending on their study condition, and install it on their home computers. Neither youth, parent or assessment staff will know what CBM version is installed; all versions appear indistinguishable to users. Youth are asked to ideally complete three intervention sessions per week over a four-week period (a goal of 12 sessions total). Youth in all three study arms may receive non-research Treatment as Usual (TAU) for anxiety and other conditions, as delivered by the HMO or other healthcare providers. TAU may include CBT, other psychotherapies, and/or medications. We will collect data on receipt of TAU from the HMO’s EHR at the study conclusion, augmented with information about any services the youth may have received outside of the HMO during the follow-up assessments.

**Fig. 2:**
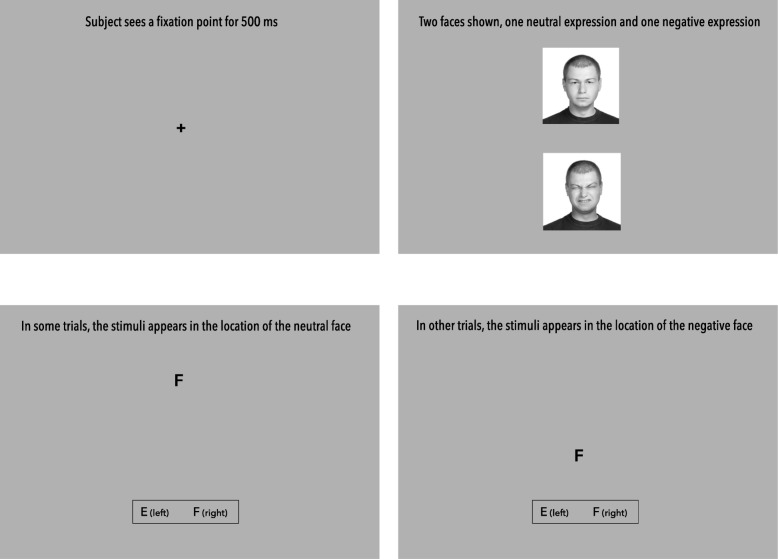
Cognitive Bias Modification (CBM) program screenshots

CBM procedures. The Low-Ratio CBM condition (Arm 1), and the two High-Ratio CBM conditions (Arms 2 and 3) share a common protocol for the delivery of the intervention. After eligibility is determined from the baseline assessment and the participant is randomized, they will be given a link and unique code to install the internet-based CBM program on their home computer. After registering with the program, the youth will complete a practice session over the phone with a study staff member. The same set of stimuli will be used for all three conditions, making the downloaded program appear identical to all users. Each youth will be instructed to complete three 15-min sessions per week for four weeks (12 sessions total). In each session, youth view 160 trials of a computerized probe detection task (simulated screenshots in Fig. 2). In each trial, two faces are briefly displayed one above the other, from a standardized set of 8 individuals. For all three CBM conditions, 80% of trials consist of neutral and negative (disgusted) face pairs, while the remaining 20% of trials consist of two neutral faces. Following a 500 ms presentation, the images disappear and a letter probe (either “E” or “F”) is displayed in the location where either the top or bottom face had just been. Youth are instructed to decide whether the letter was an “E” or an “F” with a left or right click of a mouse button. Youth do not receive feedback indicating the accuracy of their responses. In the 80% of High Ratio CBM trials (Arms 2, 3) where a neutral and disgusted face are present, the probe (E or F) always replaces the neutral face. In this manner, youth are trained to disengage their attention from threat. In the Low-Ratio condition (Arm 1), the probe replaces the neutral and disgusted faces with equal frequency (50–50%) in the trials containing both types of faces, and thus only minimal threat retraining occurs. The same library of stimuli is used for all training sessions. Images of faces will be used, rather than emotional words, because facial stimuli have been used in previous CBM studies with youths [24, 25, 61] and may control for differences in youths’ reading ability. Moreover, faces are the most common stimuli in CBM, across disorder type [62].

Phone Coach Adherence Promotion (AP) (Arm 3 Only). AP procedures are intended to mimic the effects of the nonspecific, yet likely important, support and instruction provided by research staff when CBM has been traditionally delivered in laboratories. This includes technical assistance with use of the program, support/encouragement, motivational enhancement, and brainstorming solutions to barriers to regular sessions. We hypothesize that the addition of AP will lead to greater participant adherence to the program and therefore better clinical outcomes. In the first 4 weeks of each youth’s enrollment, phone coaches will make between one and four brief, 10–30 min AP telephone calls to youth enrolled in the High-Ratio CBM + AP condition (Arm 3). Coaches may also involve parents as appropriate. Call frequency will be determined by need, primarily based on the participant’s level of adherence to the CBM program. Participants who complete at least 80% of required sessions with a accuracy rate of 80% or higher will be considered adherent to the program. Youth who are most adherent to the protocol may receive only one or two AP calls from their coach, whereas youth with inconsistent or suboptimal use of the CBM program will receive more calls, up to a total of four. Phone coaches will receive automated adherence reports by logging into the server that stores the session data (e.g., number of trials, accuracy, reaction time). Phone coaches will use these reports to determine the need for AP calls, and the type of coaching they will provide (e.g., low accuracy rate and short reaction time might suggest that youth should slow down slightly to be more accurate). Calls do not need to be once weekly, but may be concentrated (e.g., twice weekly for the first few weeks) in order to facilitate participant engagement and maximize exposure to the CBM program. Coaches will record the date, duration, and content addressed in each call.

The phone coaches will provide the explicit rationale and instructions for the CBM intervention [29], as well as the “fear priming” instructions which encourage youth to activate anxiety fears prior to each CBM session to potentiate the effectiveness of CBM skills training [63]. The remaining content of phone coach calls will be individualized to the needs/issues of each participant, but will typically address: technical assistance (difficulty downloading CBM program, registering, creating a user account); encouragement and reinforcement for appropriate use; problem solving to overcome barriers and obstacles to use; using brief motivational enhancement techniques to encourage participants to use the program [30–32]; and/or coaching and advice to ensure high quality CBM sessions (e.g., conducting sessions at times/locations where participants will not be interrupted). If needed, phone coaches will also provide case-management and referrals for emergent clinical issues not addressed by CBM program (e.g., suicidality), although they will explicitly not themselves provide any psychosocial therapy for anxiety or any other issues.

**CBM Session review** questions will be asked of youth at 1-month follow-up. Questions ask about the number and length of sessions completed, which device was used, and interruptions during session completion.

### Treatment adherence and process data

CBM adherence data is also recorded automatically by the CBM program as part of its delivery across all arms. Number of sessions is calculated as the number of sessions completed from baseline to the 1-month follow-up. A session is defined as at least 160 completed trials. Quality of participation includes: missing data due to non-response, inaccurate responses (pressing random buttons), and probe response latencies outside of the task window (< 200 ms, > 3500 ms). As a check on the adequacy of our overall CBM implementation, we will benchmark the quality of participant performance against the CBM literature, for the total sample and by arm. Number of trainings and participation quality are also secondary outcomes, with the hypothesis that the Adherence Promotion arm (Arm 3) will show superior effects on anxiety due to an expected higher number of CBM sessions.

### Reliability ratings

#### Analysis plan

##### Data collection

At each telephone assessment research evaluation staff directly enter participant responses to interviews (e.g., the ADIS) or clinician rating forms (e.g., the PARS) into a RedCap data entry system. For later follow-up assessments, participants are given an opportunity to complete self-report measures directly on a RedCap survey page. Entered data values are limited to permitted ranges. This RedCap system is installed on the research Center data servers and receives daily backup along with all other research documents/databases.

##### Preliminary data evaluation

Prior to conducting data analyses, we will audit the data for completeness and quality, including missing data. We will evaluate distributions to ensure that they meet assumptions of planned analyses (see below), including the detection of outliers.

We will use a two-tailed α = .05 for the analyses of the primary outcomes. For the secondary outcomes, we will account for multiple testing by controlling the false discovery rate (10%) using the Benjamini–Hochberg procedure. All analyses will be carried out using the intent-to-treat principle by including all participants in analyses and their original arm assignments.

##### Statistical analysis

For each continuous outcome (anxiety symptoms [PARS, SCARED], depression [PHQ-9], psychopathology severity [CGI-S], psychosocial functioning [CGAS]) we will test whether there are differences between arms in the trajectories across time by fitting growth curve models in a nested two-level hierarchical linear model (HLM [64–66];).We will test the primary outcome (anxiety remission based on ADIS-C) and the CGI-I with a generalized form of HLM, using a logit link and the binomial distribution. For all HLM models, the first level of the model represents within-person variation and will include time as a predictor (3–5 timepoints, depending on the measure). The second model level represents between-person variation and includes dummy indicators for arm as the predictor variables. A significant coefficient for arm on the slope of time indicates (i.e., the cross-level interaction of arm by time) that there are different trajectories across time for each arm and will be followed with a graph to interpret the interaction(s). Assuming that the trajectories are nonlinear as is common in clinical trials (e.g., initial intervention effect upon completing the intervention and maintenance period), we will use fractional polynomials to estimate the most appropriate function for time [67, 68]. We will test our primary hypotheses using linear contrasts of the dummy vectors representing treatment arm to determine whether both High-Ratio CBM arms (2 and 3) have a different trajectory than the Low-Ratio arm, and whether High-Ratio CBM + AP (Arm 3) has a different trajectory than High-Ratio CBM Only (Arm 2). Differences at 1 month in acceptability (PAQ) and client satisfaction (CSQ-8) will be tested with a one-way ANOVA with treatment arm as the independent variable and the same a priori contrasts described above.

We will conduct the moderator analyses by adding the moderator and moderatorXarm product in the level-two HLM equation discussed above. A significant coefficient for the interaction term on the slope of time would indicate a moderator effect and will be followed up with graphs of the simple effects to determine the nature of the effect modification.

We will test whether attention bias mediates between treatment and the primary outcome (remission status), by regressing Wk 2 attention bias on attention bias at baseline and study arm indicators, and by regressing remission status (using logistic regression) at one month on attention bias at 2 weeks and study arm indicators. Using the product coefficients approach to mediation [69–71], we will use the resulting regression coefficients and standard errors (SEs) to estimate the indirect effect. Because SEs of the products of regression coefficients are not normal, we will use bias-corrected bootstrapping to estimate the SEs for the indirect effect [72].

##### Missing data

Despite best efforts, we expect attrition of ~ 10% by 1 month and ~ 15% by 12 months. We will handle missing panel data using direct maximum likelihood (ML) [73–75], which uses all available data provided by a given participant. ML, like multiple imputation, can provide valid inferences when data are “missing at random” and are superior to other methods which are limited to a more stringent assumption of “missing completely at random.”

##### Power analyses

Preliminary to calculations, we conducted a review of the adult and youth CBM anxiety literature to estimate plausible effect sizes. This included the five adult RCTs [18–22], two youth RCTs, [23, 25] and one youth case series [24]. To increase comparability across studies, we calculated within-condition effect sizes (ESs) for anxiety reduction associated with the variants of CBM most relevant for this proposal. For Low-Ratio CBM, we found a mean ES of d = 0.52. For self-administered CBM (often Internet delivered), we found a mean ES of 0.82. Lab-administered CBM yielded a mean ES of 1.39. Finally, CBM with a mix of lab and self-administered sessions yielded a mean ES similar to lab-based studies (mean d = 1.56). From this review we (a) projected the within- and between-condition ES differences we might expect in our proposed design and (b) calculated the effective ES for each of our hypotheses of clinical outcomes. For the comparison of the two High-Ratio CBM conditions vs. Low-Ratio CBM, our review suggests an estimated difference (Δ) ES of d = 0.73. We conservatively powered this analysis for an ES Δ of 0.59, approximately equivalent to an odds ratio of 2.90 [76]. To estimate the ES for the comparison of CBM + AP and High-Ratio CBM, we looked to the literature for estimates of the comparison of lab-administered vs. self-administered CBM. This review suggests an estimated ES Δ of d = 0.57. We chose to power the analysis for this hypothesis with a conservative ES Δ of d = 0.37, which is approximately equivalent to an odds ratio of 1.94 [76].

We conducted power analyses for the primary outcome (via PASS software [77]) to generate the sample required to detect the hypothesized contrasts in a logistic regression model. For our main comparison of the two High-Ratio CBM conditions versus Low-Ratio CBM, with α = .05, we would have 80% power to detect a 20% greater remission (odds ratio = 2.90) in arms 2 + 3 compared to the Low-Ratio arm with 187 participants (n~ 63 per arm) at the 6-month follow-up**.** For the comparison of the two High-Ratio CBM conditions, at an alpha level of .05, we would have 80% power to detect a 16% greater remission (odds ratio = 1.94) in the CBM + AP arm compared to the CBM only arm with 148 participants per arm (*N* = 444 total) at the 6-month follow-up. We based our target sample on the larger N needed to adequately test this latter hypothesis (Arm 2 vs. Arm 3). To compensate for the loss in precision from imputing data for those lost to follow-up, we inflated the target sample size based on an attrition rate of ~ 10% (*N* = 498, or *n* = 166 per arm). This is a conservative adjustment, as the SEs from imputed data results will be less than the SEs from a complete case analysis.

##### Economic analyses

We will conduct incremental cost-effectiveness analyses (CEA) following the methodology recommended by the Panel on Cost-Effectiveness in Health & Medicine [78] and methods used in prior CEAs [79, 80]. For our primary CEA, we will take the health-care organization perspective for 12 months following randomization, calculated from the EQ-5D as the clinical outcome because this allows the broadest comparison to other medical interventions, and is recommended by experts [78]. Although other perspectives, such as the societal perspective, may be important to understand, the healthcare perspective is particularly relevant when the focus is on the healthcare system implementing the intervention. The EQ-5D has been used in numerous previous CEAs, including several with anxiety interventions [81–83]. However, QALYs may not capture all relevant mental health clinical impacts [84–86]. Thus, we will also calculate incremental CEAs using anxiety-free days (AFD) similar to previous anxiety treatment CEAs [81–83, 87–89]. AFDs are one of the most commonly employed outcomes in economic analyses of anxiety treatments [87, 90–93], enabling us to compare our economic results with the rest of the field. As part of the economic evaluation, we will also use instrumental variable methods to attempt to better estimate the effect of CBM dose on QALYs and cost, using potential instruments collected as part of the baseline assessment.

## Discussion

This paper describes the study protocol of a randomized controlled trial (RCT) to assess the benefit of computerized CBM to treat youth with anxiety. This RCT is considered a blended efficacy-effectiveness trial, in that it is highly controlled with detailed, blinded assessments and has a focus on possible treatment mechanisms, but is also conducted in a real-life setting with youth who have sought usual care services for anxiety and who have received a clinical anxiety diagnosis. The primary aim of this study is to assess whether self-administered CBM can be successfully delivered as a self-administered treatment at home, and whether High-Ratio CBM will demonstrate clinical effectiveness. Further, we aim to assess whether High-Ratio CBM with the AP component will result in greater rates of anxiety remission than High-Ratio CBM without AP. Finally, we aim to assess whether self-administered CBM is cost effective to deliver to youth seeking anxiety treatment in a real-world health-care setting.

### Strengths

The study uses a rigorous control condition with a Low-Ratio CBM design, which controls for the effects of time and attention. The youth in all three arms will be asked to complete the same number of sessions over the intervention period, and in each session the youth view the same number of trials. These features bring the rigor of a classic efficacy design and permit confident attributions of outcome effects.

This study will have “effectiveness” elements (such as recruitment via electronic medical records and self-administration of CBM), which will allow us to enroll a sample that is 10 times larger than other published CBM trials. This study will be large enough to have the statistical power to pursue a wide variety of substantive, mechanistic, and economic analyses that smaller lab-based studies could not address. However, the sample will not be so large that we are unable to pursue a detailed evaluation of clinical outcomes.

### Limitations

Although self-administered CBM promises to be an easily disseminated treatment for anxiety, past results have been mixed when CBM has been delivered outside of the lab. It is possible that without any in-person staff support, participants will not adhere to the protocol as closely as if they were in the lab. However, the design of the current study has attempted to address this potential limitation by adding the telephone-based adherence promotion arm to address these potential issues, which will be the first to test the importance of this component. This added component will add additional support provided by research staff to give technical assistance with the use of the program, support/encouragement, motivational enhancement, and brainstorming solutions to barriers. The addition of AP in this trial will attempt to recreate some of the support of in-person lab-based interventions, which we hypothesize will lead to greater clinical effectiveness of the self-administered CBM program.

Another potential limitation is that the control condition is itself an active treatment, albeit of a lower “dose” of negative bias retraining opportunities. While this controls for expectation bias and treatment exposure, it also may prove to be a more a more difficult control condition for the High-Ratio CBM to overcome.

The study is a mixed-blind design, where participants are unaware whether they are being administered the High- or Low-Ratio version of the CBM program. However, with the addition of the AP component in Arm 3, it is impossible for the participants and phone coaches in this arm to be blind to their condition. To address this, we have taken measures to ensure that the research evaluation staff remain blinded to participant condition and complete assessments without knowledge of their assignment, including whether a given participant is receiving AP.

Our planned sample will have approximately 141 participants in each arm at 12-month follow-up, accounting for an expected attrition rate of 10%. A sample this size is sufficient to detect expected clinical differences, but will give us a less than optimal level of power to detect differences in the cost effectiveness analyses which ideally compare hundreds or thousands of cases per condition. Although this is a limitation, the economic evaluation will still provide useful information about the likely effect size related to cost effectiveness for the intervention and this information could help refine the intervention in order to increase its potential for implementation in other health-care settings.

This self-administered CBM intervention overcomes barriers to EBTs for anxiety such as cognitive behavioral therapy and SSRI medication, as well as limitations to previous studies that require participants to attend a centrally located clinic and complete sessions in a lab. The at-home format of this intervention makes it uniquely easy to disseminate, and this study will fill a gap in the literature by comparing the costs and outcomes of delivering CBM both with and without an adherence promotion component. This will provide the information needed to consider whether broad dissemination and implementation of this intervention is feasible.

## Data Availability

Not applicable.

## Abbreviations

AD: Antidepressant
AFD: Anxiety-free day
AFD: Anxiety-free day
AP: Adherence promotion
CBM: Cognitive bias modification
CBT: Cognitive behavioral therapy
DSM: Diagnostic and Statistical Manual
EBTs: Evidence-based treatments
EHR: Electronic health record
GAD: General anxiety disorder
HLM: Hierarchical linear model
HMO: Health maintenance organization
IRB: Institutional Review Board
KPNW: Kaiser Permanente Northwest
ML: Maximum likelihood
QALY: Quality adjusted life year
RCT: Randomized controlled trial
SEs: Standard errors
SP: Social phobia
SSRIs: Selective Serotonin Reuptake Inhibitors
TAU: Treatment as usual

## References

[1] Merikangas KR, He JP, Burstein M, Swanson SA, Avenevoli S, Cui L (2010). Lifetime prevalence of mental disorders in U.S. adolescents: results from the National Comorbidity Survey Replication--Adolescent Supplement (NCS-A). J Am Acad Child Adolesc Psychiatry.

[2] Kessler RC, Chiu WT, Demler O, Merikangas KR, Walters EE (2005). Prevalence, severity, and comorbidity of 12-month DSM-IV disorders in the National Comorbidity Survey Replication. Arch Gen Psychiatry.

[3] Marciniak MD, Lage MJ, Dunayevich E, Russell JM, Bowman L, Landbloom RP (2005). The cost of treating anxiety: the medical and demographic correlates that impact total medical costs. Depress Anxiety..

[4] Revicki DA, Travers K, Wyrwich KW, Svedsater H, Locklear J, Mattera MS (2012). Humanistic and economic burden of generalized anxiety disorder in North America and Europe. J Affect Disord.

[5] Bandelow B, Michaelis S (2015). Epidemiology of anxiety disorders in the 21st century. Dialogues Clin Neurosci.

[6] Harvard Medical School. National Comorbidity Survey (NCS) Data Table 2: 12-month prevalence DSM-IV/WMH-CIDI disorders by sex and cohort. 2007. https://www.hcp.med.harvard.edu/ncs/ftpdir/table_ncsr_12monthprevgenderxage.pdf. 2019.

[7] Harvard Medical School. National Comorbidity Survey (NCS) Data Table 1: Lifetime prevalence DSM-IV/WMH-CIDI disorders by sex and cohort. 2007. https://www.hcp.med.harvard.edu/ncs/ftpdir/table_ncsr_LTprevgenderxage.pdf. 2019.

[8] Merikangas KR, He JP, Brody D, Fisher PW, Bourdon K, Koretz DS (2010). Prevalence and treatment of mental disorders among US children in the 2001-2004 NHANES. Pediatrics..

[9] Ringle VA, Read KL, Edmunds JM, Brodman DM, Kendall PC, Barg F (2015). Barriers to and facilitators in the implementation of cognitive-behavioral therapy for youth anxiety in the community. Psychiatr Serv.

[10] Hallerman Price J. Adherence Barriers to Antidepressants among an Urban Female Latino Population 2013. https://ethnomed.org/clinical/mental-health/adherence-barriers-to-antidepressants-among-an-urban-female-latino-population. 2019.

[11] Mathews A, MacLeod C (2005). Cognitive vulnerability to emotional disorders. Annu Rev Clin Psychol.

[12] MacLeod Colin, Grafton Ben, Notebaert Lies (2019). Anxiety-Linked Attentional Bias: Is It Reliable?. Annual Review of Clinical Psychology.

[13] Bar-Haim Y, Lamy D, Pergamin L, Bakermans-Kranenburg MJ, Van Ijzendoorn MH (2007). Threat-related attentional bias in anxious and nonanxious individuals: a meta-analytic study. Psychol Bull.

[14] Clarke PJ, Notebaert L, MacLeod C (2014). Absence of evidence or evidence of absence: reflecting on therapeutic implementations of attentional bias modification. BMC Psychiatry..

[15] Sportel BE, de Hullu E, de Jong PJ, Nauta MH (2013). Cognitive bias modification versus CBT in reducing adolescent social anxiety: a randomized controlled trial. PLoS One.

[16] Taylor C, Aupperle R, Flagan T. al. e. Attentional training boosts top-down neural activation underlying emotion processing in anxious subjects. Biol Psychiatry. In Press.

[17] Koster EH, Fox E, MacLeod C (2009). Introduction to the special section on cognitive bias modification in emotional disorders. J Abnorm Psychol.

[18] Amir N, Beard C, Burns M, Bomyea J (2009). Attention modification program in individuals with generalized anxiety disorder. J Abnorm Psychol.

[19] Schmidt NB, Richey JA, Buckner JD, Timpano KR (2009). Attention training for generalized social anxiety disorder. J Abnorm Psychol.

[20] Amir N, Beard C, Taylor CT, Klumpp H, Elias J, Burns M (2009). Attention training in individuals with generalized social phobia: a randomized controlled trial. J Consult Clin Psychol.

[21] Heeren A, Reese HE, McNally RJ, Philippot P (2012). Attention training toward and away from threat in social phobia: effects on subjective, behavioral, and physiological measures of anxiety. Behav Res Ther.

[22] Carlbring P, Apelstrand M, Sehlin H, Amir N, Rousseau A, Hofmann SG (2012). Internet-delivered attention bias modification training in individuals with social anxiety disorder--a double blind randomized controlled trial. BMC Psychiatry.

[23] Riemann BC, Kuckertz JM, Rozenman M, Weersing VR, Amir N (2013). Augmentation of youth cognitive behavioral and pharmacological interventions with attention modification: a preliminary investigation. Depress Anxiety.

[24] Rozenman M, Weersing VR, Amir N (2011). A case series of attention modification in clinically anxious youths. Behav Res Ther.

[25] Eldar S, Apter A, Lotan D, Edgar KP, Naim R, Fox NA (2012). Attention bias modification treatment for pediatric anxiety disorders: a randomized controlled trial. Am J Psychiatry.

[26] Ioannidis JP (2005). Contradicted and initially stronger effects in highly cited clinical research. Jama..

[27] Neubauer K, von Auer M, Murray E, Petermann F, Helbig-Lang S, Gerlach AL (2013). Internet-delivered attention modification training as a treatment for social phobia: a randomized controlled trial. Behav Res Ther.

[28] Beard C, Sawyer AT, Hofmann SG (2012). Efficacy of attention bias modification using threat and appetitive stimuli: a meta-analytic review. Behav Ther.

[29] Krebs G, Hirsch CR, Mathews A (2010). The effect of attention modification with explicit vs. minimal instructions on worry. Behav Res Ther.

[30] Digdon NL, Howell AJ (2008). College students who have an eveningness preference report lower self-control and greater procrastination. Chronobiol Int.

[31] Hettema J, Steele J, Miller WR (2005). Motivational interviewing. Annu Rev Clin Psychol.

[32] Miller WR, Rollnick S (2012). Motivational interviewing: preparing people for change. Second edition ed.

[33] Amir N, Taylor CT, Donohue MC (2011). Predictors of response to an attention modification program in generalized social phobia. J Consult Clin Psychol.

[34] Silverman WK, Saavedra LM, Pina AA (2001). Test-retest reliability of anxiety symptoms and diagnoses with the anxiety disorders interview schedule for DSM-IV: child and parent versions. J Am Acad Child Adolesc Psychiatry.

[35] Silverman WK, Nelles WB (1988). The anxiety disorders interview schedule for children. J Am Acad Child Adolesc Psychiatry.

[36] RUPP (2002). The pediatric anxiety rating scale (PARS): development and psychometric properties. J Am Acad Child Adolesc Psychiatry.

[37] Compton SN, Walkup JT, Albano AM, Piacentini JC, Birmaher B, Sherrill JT (2010). Child/adolescent anxiety multimodal study (CAMS): rationale, design, and methods. Child Adolesc Psychiatry Ment Health.

[38] Walkup JT, Albano AM, Piacentini J, Birmaher B, Compton SN, Sherrill JT (2008). Cognitive behavioral therapy, sertraline, or a combination in childhood anxiety. N Engl J Med.

[39] Birmaher B, Brent DA, Chiappetta L, Bridge J, Monga S, Baugher M (1999). Psychometric properties of the screen for child anxiety related emotional disorders (SCARED): a replication study. J Am Acad Child Adolesc Psychiatry.

[40] Whiteside SP, Gryczkowski M, Ale CM, Brown-Jacobsen AM, McCarthy DM (2013). Development of child- and parent-report measures of behavioral avoidance related to childhood anxiety disorders. Behav Ther.

[41] Richardson LP, McCauley E, Grossman DC, McCarty CA, Richards J, Russo JE (2010). Evaluation of the patient health Questionnaire-9 item for detecting major depression among adolescents. Pediatrics..

[42] Guy W (1976). Clinical global improvement scale.

[43] Shaffer D, Gould MS, Brasic J, Ambrosini P, Fisher P, Bird H (1983). A children's global assessment scale (CGAS). Arch Gen Psychiatry.

[44] Ascher BHZ, Farmer EM, Burns BJ, Angold A (1996). The child and adolescent services assessment (CASA) description and psychometrics. J Emot Behav Disord.

[45] EuroQol G (1990). EuroQol--a new facility for the measurement of health-related quality of life. Health Policy.

[46] Rabin R, de Charro F (2001). EQ-5D: a measure of health status from the EuroQol group. Ann Med.

[47] Bereza BG, Machado M, Einarson TR (2009). Systematic review and quality assessment of economic evaluations and quality-of-life studies related to generalized anxiety disorder. Clin Ther.

[48] Saarni SI, Suvisaari J, Sintonen H, Pirkola S, Koskinen S, Aromaa A (2007). Impact of psychiatric disorders on health-related quality of life: general population survey. Br J Psychiatry.

[49] Whynes DK (2009). Responsiveness of the EQ-5D to HADS-identified anxiety and depression. J Eval Clin Pract.

[50] Willems DC, Joore MA, Nieman FH, Severens JL, Wouters EF, Hendriks JJ (2009). Using EQ-5D in children with asthma, rheumatic disorders, diabetes, and speech/language and/or hearing disorders. Int J Technol Assess Health Care.

[51] Julian LJ (2011). Measures of anxiety: state-trait anxiety inventory (STAI), Beck anxiety inventory (BAI), and hospital anxiety and depression scale-anxiety (HADS-A). Arthritis Care Res (Hoboken).

[52] Carskadon MA, Acebo C (1993). A self-administered rating scale for pubertal development. J Adolesc Health.

[53] Brooks-Gunn J, Warren MP, Rosso J, Gargiulo J. Validity of self-report measures of girls' pubertal status. Child Dev. 1987. 3608653

[54] Petersen AC, Crockett L, Richards M, Boxer A (1988). A self-report measure of pubertal status: reliability, validity, and initial norms. J Youth Adolescence.

[55] Bastien CH, Vallieres A, Morin CM (2001). Validation of the insomnia severity index as an outcome measure for insomnia research. Sleep Med.

[56] Morin C. M. Insomnia: psychological assessment and management: Guilford press; 1993.

[57] Attkisson CC, Greenfield T (1995). The client satisfaction questionnaire (CSQ) scales. Outcome assessment in clinical practice.

[58] Mander J, Wittorf A, Teufel M, Schlarb A, Hautzinger M, Zipfel S (2012). Patients with depression, somatoform disorders, and eating disorders on the stages of change: validation of a short version of the URICA. Psychotherapy (Chic).

[59] Dunn G, Bentall R (2007). Modelling treatment-effect heterogeneity in randomized controlled trials of complex interventions (psychological treatments). Stat Med.

[60] Wells KB, Tang L, Carlson GA, Asarnow JR (2012). Treatment of youth depression in primary care under usual practice conditions: observational findings from youth Partners in Care. J Child Adolesc Psychopharmacol.

[61] Eldar S, Ricon T, Bar-Haim Y (2008). Plasticity in attention: implications for stress response in children. Behav Res Ther.

[62] Bar-Haim Y (2010). Research review: attention bias modification (ABM): a novel treatment for anxiety disorders. J Child Psychol Psychiatry.

[63] Kuckertz JM, Gildebrant E, Liliequist B, Karlström P, Väppling C, Bodlund O (2014). Moderation and mediation of the effect of attention training in social anxiety disorder. Behav Res Ther.

[64] Raudenbush S. W., Bryk A. S. Hierarchical linear models: applications and data analysis methods: sage; 2002.

[65] Snijders TBR (1999). Multilevel analysis: an introduction to basic and advanced multilevel modeling.

[66] Hox J. J., Moerbeek M., Van de Schoot R. Multilevel analysis: techniques and applications: Routledge; 2017.

[67] Royston P, Altman DG (1994). Regression using fractional polynomials of continuous covariates: parsimonious parametric modelling. J R Stat Soc: Ser C: Appl Stat.

[68] Royston P, Sauerbrei W (2008). Multivariable model-building: a pragmatic approach to regression anaylsis based on fractional polynomials for modelling continuous variables: John Wiley & Sons.

[69] Hayes AF (2009). Beyond baron and Kenny: statistical mediation analysis in the new millennium. Commun Monogr.

[70] MacKinnon D. Introduction to statistical mediation analysis: Routledge; 2012.

[71] MacKinnon DP, Lockwood CM, Hoffman JM, West SG, Sheets V (2002). A comparison of methods to test mediation and other intervening variable effects. Psychol Methods.

[72] Mackinnon DP, Lockwood CM, Williams J (2004). Confidence limits for the indirect effect: distribution of the product and resampling methods. Multivariate Behav Res.

[73] Enders C. K. Applied missing data analysis: Guilford press; 2010.

[74] Schafer JL, Graham JW (2002). Missing data: our view of the state of the art. Psychol Methods.

[75] Allison P. D. Missing data: sage thousand oaks, CA; 2010.

[76] Chinn S (2000). A simple method for converting an odds ratio to effect size for use in meta-analysis. Stat Med.

[77] Hintze J (2008). PASS 2008.

[78] Weinstein M. C., Russell L. B., Gold M. R., Siegel J. E. Cost-effectiveness in health and medicine: Oxford university press; 1996. 8849754

[79] Lynch FL, Striegel-Moore RH, Dickerson JF, Perrin N, Debar L, Wilson GT (2010). Cost-effectiveness of guided self-help treatment for recurrent binge eating. J Consult Clin Psychol.

[80] Lynch FL, Dickerson JF, Clarke G, Vitiello B, Porta G, Wagner KD (2011). Incremental cost-effectiveness of combined therapy vs medication only for youth with selective serotonin reuptake inhibitor-resistant depression: treatment of SSRI-resistant depression in adolescents trial findings. Arch Gen Psychiatry.

[81] Konig HH, Born A, Heider D, Matschinger H, Heinrich S, Riedel-Heller SG (2009). Cost-effectiveness of a primary care model for anxiety disorders. Br J Psychiatry.

[82] Hoek W, Schuurmans J, Koot HM, Cuijpers P (2009). Prevention of depression and anxiety in adolescents: a randomized controlled trial testing the efficacy and mechanisms of internet-based self-help problem-solving therapy. Trials..

[83] Bodden DH, Dirksen CD, Bogels SM, Nauta MH, De Haan E, Ringrose J (2008). Costs and cost-effectiveness of family CBT versus individual CBT in clinically anxious children. Clin Child Psychol Psychiatry.

[84] Chisholm D, Healey A, Knapp M (1997). QALYs and mental health care. Soc Psychiatry Psychiatr Epidemiol.

[85] Knapp M., Mangalore R. "The trouble with QALYs...". Epidemiol Psichiatr Soc 2007; 16(4):289–293. 10.1017/s1121189x00002451 18333423

[86] Brazier J (2008). Measuring and valuing mental health for use in economic evaluation. J Health Serv Res Policy.

[87] Iskedjian M, Walker JH, Bereza BG, Le Melledo JM, Einarson TR (2008). Cost-effectiveness of escitalopram for generalized anxiety disorder in Canada. Curr Med Res Opin.

[88] McCrone P, Knapp M, Proudfoot J, Ryden C, Cavanagh K, Shapiro DA (2004). Cost-effectiveness of computerised cognitive-behavioural therapy for anxiety and depression in primary care: randomised controlled trial. Br J Psychiatry.

[89] Simon E, Dirksen C, Bogels S, Bodden D (2012). Cost-effectiveness of child-focused and parent-focused interventions in a child anxiety prevention program. J Anxiety Disord.

[90] Katon WJ, Roy-Byrne P, Russo J, Cowley D (2002). Cost-effectiveness and cost offset of a collaborative care intervention for primary care patients with panic disorder. Arch Gen Psychiatry.

[91] Katon W, Russo J, Sherbourne C, Stein MB, Craske M, Fan MY (2006). Incremental cost-effectiveness of a collaborative care intervention for panic disorder. Psychol Med.

[92] Zimovetz EA, Wolowacz SE, Classi PM, Birt J (2012). Methodologies used in cost-effectiveness models for evaluating treatments in major depressive disorder: a systematic review. Cost Eff Resour Alloc.

[93] Joesch JM, Sherbourne CD, Sullivan G, Stein MB, Craske MG, Roy-Byrne P (2012). Incremental benefits and cost of coordinated anxiety learning and management for anxiety treatment in primary care. Psychol Med.

